# Dietary α-Mangostin Provides Protective Effects against Acetaminophen-Induced Hepatotoxicity in Mice via Akt/mTOR-Mediated Inhibition of Autophagy and Apoptosis

**DOI:** 10.3390/ijms19051335

**Published:** 2018-05-01

**Authors:** Xiao-tong Yan, Yin-shi Sun, Shen Ren, Li-chun Zhao, Wen-cong Liu, Chen Chen, Zi Wang, Wei Li

**Affiliations:** 1 College of Chinese Medicinal Materials, Jilin Agricultural University, Changchun 130118, China; yanxiaotong0707@126.com (X.-t.Y.); sunyinshi2002@163.com (Y.-s.S.); rs0109@163.com (S.R.); jwlw6803@126.com (W.-c.L.); wangzi8020@126.com (Z.W.); 2 Institute of Special Wild Economic Animals and Plant, CAAS, Changchun 132109, China; 3 College of Pharmacy, Guangxi University of Chinese Medicine, Nanning 530011, China; hyzlc@126.com; 4 School of Biomedical Sciences, University of Queensland, Brisbane 4072, Australia; chen.chen@uq.edu.au

**Keywords:** α-mangostin, acetaminophen, acute liver injury, apoptosis, autophagy

## Abstract

Acetaminophen overdose-induced hepatotoxicity is the most common cause of acute liver failure in many countries. Previously, alpha-mangostin (α-MG) has been confirmed to exert protective effects on a variety of liver injuries, but the protective effect on acetaminophen-induced acute liver injury (ALI) remains largely unknown. This work investigated the regulatory effect and underlying cellular mechanisms of α-MG action to attenuate acetaminophen-induced hepatotoxicity in mice. The increased serum aminotransferase levels and glutathione (GSH) content and reduced malondialdehyde (MDA) demonstrated the protective effect of α-MG against acetaminophen-induced hepatotoxicity. In addition, α-MG pretreatment inhibited increases in tumor necrosis factor (TNF-α) and interleukin-1β (IL-1β) caused by exposure of mice to acetaminophen. In liver tissues, α-MG inhibited the protein expression of autophagy-related microtubule-associated protein light chain 3 (LC3) and BCL2/adenovirus E1B protein-interacting protein 3 (BNIP3). Western blotting analysis of liver tissues also proved evidence that α-MG partially inhibited the activation of apoptotic signaling pathways via increasing the expression of Bcl-2 and decreasing Bax and cleaved caspase 3 proteins. In addition, α-MG could in part downregulate the increase in p62 level and upregulate the decrease in p-mTOR, p-AKT and LC3 II /LC3 I ratio in autophagy signaling pathways in the mouse liver. Taken together, our findings proved novel perspectives that detoxification effect of α-MG on acetaminophen-induced ALI might be due to the alterations in Akt/mTOR pathway in the liver.

## 1. Introduction

Drug-induced liver injury (DILI) is a major problem resulting in acute liver failure (ALF) in drug development and clinical application all over the world [1], in particular, acetaminophen (APAP) severely threatens human health [2]. It is accepted that APAP is widely used as an antipyretic analgesic at therapeutic doses [3]. However, supratherapeutic misuse, non-intentional misuse, and intentional ingestion may all result in hepatic toxicity, the main cause of ALF in the United States and Europe [4]. Initially, cytochrome P450 (CYP) is responsible for APAP bio-transformation into a toxic, highly biologically reactive intermediate, N-acetyl-p-aminophenol (NAPQI). Excessive NAPQI can deplete hepatic GSH and bind to proteins and DNA, which ultimately leads to hepatocyte pathological changes including oxidative stress, mitochondrial dysfunction, cellular necrosis, and apoptosis [5].

So far, N-acetyl-l-cysteine (NAC) is the foremost therapy for treating APAP-induced ALF. However, liver transplantation is often needed if patients are not treated in time [6]. Therefore, it is urgent to explore novel candidates to prevent and treat APAP-induced hepatotoxicity. The medicinal benefits of plants for protecting tissues from toxicity have been recognized for centuries. Based on their potential efficiency, low toxicity, and few side effects, Traditional Chinese Medicine (TCM) herbs have attracted attention in this field [7].

Autophagy is tightly regulated and involved in the degradation of organelles and long-living proteins in the cells [8]. Autophagy plays an important role in cell survival as well as in the regulation of cell apoptosis and death, especially apoptosis-signaling pathways [9]. Recent reports indicated that an overdose of APAP-induced autophagy in both in vitro primary cultured mouse hepatocytes and in vivo in the mouse liver [10]. Autophagy was triggered to cause drug-induced liver injury, as demonstrated in overdose APAP treatment [11].

**Figure 1:**
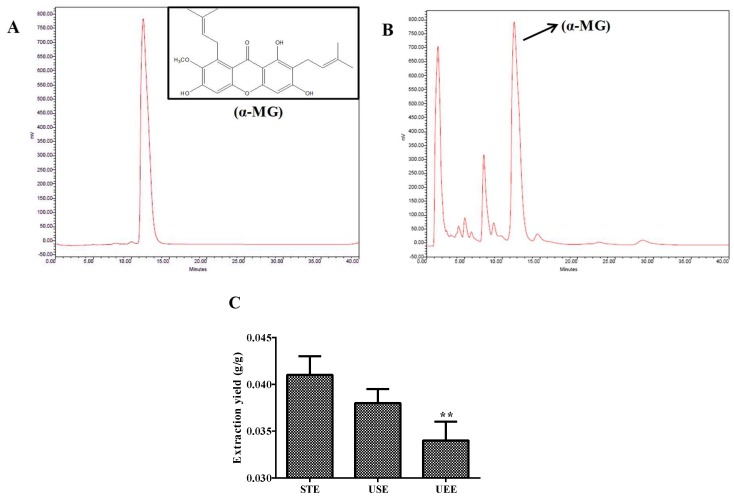
HPLC analysis of an extract obtained by STE of mangosteen pericarp. (**A**) The standard of α-mangostin; (**B**) α-mangostin in the HPLC; (**C**) comparison of different extraction techniques. All data are expressed as mean ± S.D., *n* = 3. ** *p* < 0.01 vs. STE method.

Recently, extracts of traditional medicinal plants have shown specific health effects. Mangosteen (*Garcinia mangostana*) is a well-known tropical evergreen tree and has been used as a traditional medicine for centuries [12]. As major secondary metabolites of mangosteen, xanthones are assumed to be the major active substances. Previous studies have shown that the proportion of α-mangostin (α-MG, Figure 1) in the xanthone complex of mangosteen is high. The biological activities of α-MG have led to it being used for several decades due to it being abundant, easy to obtain, inexpensive, and biologically safe [13]. As the most important secondary metabolite in the plant, α-MG demonstrates a wide variety of pharmacological effects including anti-inflammation [14,15], anti-oxidant [16], anti-apoptosis, antibacterial [17], and anti-Alzheimer’s disease [18]. α-MG was reported to significantly ameliorate hepatic steatosis in vivo [19,20]. In addition, α-MG was shown to reduce the risk of liver fibrosis on thioacetamide-induced cirrhosis in rats [21]. Based on accumulating evidence on liver cirrhosis in vivo, it is possible that APAP-induced hepatic injury may cause further liver fibrosis. The working hypothesis here is that α-MG may have the capacity to prevent APAP-induced hepatotoxicity due to its protective effect against liver fibrosis and anti-apoptosis effect. Considering that APAP-induced hepatotoxicity can lead to inflammation, oxidative stress, autophagy, and cellular necrosis, a study of the protective effects of α-MG and relevant molecular mechanisms were undertaken.

## 2. Materials and Methods

### 2.1. Chemicals and Reagents

APAP was obtained from Sigma-Aldrich (St. Louis, MO, USA). The commercial assay kits for AST, ALT, MDA, GSH, and hematoxylin and eosin (H&E) dye kits were purchased from Nanjing Jiancheng Bioengineering Research Institute (Nanjing, China). ELISA kits for mouse TNF-α and IL-1β were obtained from R&D Systems (Minneapolis, MN, USA). The apoptosis detection kit of terminal dUTP nick-end labeling (TUNEL) staining was bought from Beyotime Biotechnology (Shanghai, China). Antibodies against rabbit proteins as anti-LC3, anti-cleaved caspase 3, anti-p-Akt, anti-p62, anti-BNIP3, and anti-mTOR were acquired from Cell Signaling Technology (Danvers, MA, USA). Antibody anti-mouse-β-actin was provided by Proteintech (Rosemont, IL, USA). Antibodies anti-mouse caspase 3, rabbit anti-Bax, and rabbit anti-Bcl-2 were obtained from Abcam (Cambridge, UK). SABC-DyLight 488 was purchased from BOSTER Biological Technology Co., Ltd. (Wuhan, China).

### 2.2. Plant Material and Preparation of α-MG from Mangosteen Pericarp

Mangosteen was purchased from Carrefour in Changchun and authenticated by Prof. Wei Li from College of Chinese Medicinal Materials, Jilin Agricultural University. Voucher specimens were deposited in the herbarium in the College of Chinese Medicinal Materials, Jilin Agricultural University, Changchun. α-MG was extracted from the pericarp of mangosteen and quantified in our laboratory. Briefly, the pericarp of the fruits was dried at 50 °C and then pulverized into a homogeneous size by a disintegrator (HX-200A, Yongkang Hardware and Medical Instrument Plant, China) and then sieved (30–40 mesh). The dried mangosteen pericarp powder (20 g) was extracted with 200 mL of 85% ethanol for 5 min, twice by smashing tissue extraction (STE). Then, the filtered solution was concentrated to dryness under a vacuum at 50 °C. The product was dissolved in 25 mL methanol, and then the supernatant was filtered through 0.45 μm nylon filter and analyzed for the quantification of α-MG content by the HPLC method. In the present study, ultrasound-assisted extraction (USE) and ultrasonic-assisted enzymatic extraction (UEE) were compared with STE on the extraction yield of α-mangostin from the pericarp of mangosteen. Under optimal conditions, the extraction yield of α-MG obtained using the above STE method was significantly higher than those using USE and UEE methods. The extraction yield of α-MG was 4.1% (Figure 1C). The purity of α-MG was determined by the HPLC method to be more than 98%. 

### 2.3. Animals and Experimental Design 

Male ICR mice (Body weight 26.2 ± 1.5 g, 8 weeks old) were obtained from YISI Experimental Animal Holding with a Certificate of Quality No. SCXK-JI-2017-0018 (Changchun, China) and maintained at 23.0 ±2.0 °C with 50–70% humidity on a 12 h light–dark cycle with free access to food and water. All experimental animals program were strictly performed according to the Guide for the Care and Use of Laboratory Animals (2016). All the animal experiments were approved by the Ethical Committee for Laboratory Animals of Jilin Agricultural University (Permit No.: ECLA-JLAU-17091).

After acclimation for one week, the mice were randomly divided into four groups (*n* = 8): normal group, APAP group, APAP and two doses of α-MG groups (100 and 200 mg/kg body weight, respectively). α-MG exact was suspended in 0.05% carboxymethylcellulose sodium (CMC-Na). α-MG was administered intragastrically to mice in α-MG groups for seven consecutive days. In the meantime, the normal and APAP groups were administered with 0.05% CMC-Na. On the sixth day, a single dose of APAP (250 mg/kg, dissolved in warm water) was injected into mice in APAP and α-MG groups after 1 h from the last administration to induce acute hepatotoxicity. All mice were killed 24 h after APAP exposure. And at the same time, the experimental mice in normal group were received with 0.05% CMC-Na. Serum samples were collected for analysis of biochemical parameters. Liver samples were collected and used for measurement of GSH content, MDA level, and Western blotting analysis. Parts of liver samples were fixed in formalin for histological examination and immunofluorescence analysis.

### 2.4. Determination of α-MG by HPLC 

The detection of α-MG content was performed using HPLC assay method as described previously with minor modifications [22]. Briefly, HPLC analysis was performed using a Waters LC system (Milford Massachusetts, USA) equipped with a Waters TM600 pump and controller, and a Waters TM2487 intelligent UV-vis detector as well as Millennium 32 system software. Chromatographic separation was performed on YMC-C18 reverse-phase column (YMC-Pack ODS-A 150 mm × 6.0 mm I.D. S-5 μm, 12 nm). The mobile phase consisted of acetonitrile–water (85:15). The UV detector was set at a wavelength of 280 nm. The flow rate was 1.0 mL/min, injection volume was 5.0 μL, and column temperature was maintained at 30 °C.

### 2.5. Analysis of Histology Changes and TUNEL Assay

For the histology examination, liver samples were fixed immediately with neutral buffered formalin solution. Liver sections (5 μm thickness) were prepared from paraffin-embedded tissue and subjected to H&E staining for further histological analysis under a light microscope (100× and 400×) [23]. The necrotic degree (as a percentage of the total area) was assessed by the necrotic area, inflammatory cell infiltration degree, and congestion relative to the entire histological sections. The results were expressed as necrosis scores.

The TUNEL assay was performed by in situ apoptosis detection kits (Beyotime Biotechnology, China) to detect hepatocyte apoptosis in liver tissues according to the manufacturer’s instructions. Firstly, the liver sections (5 μm thickness) were permeabilized by incubating with 100 μL of 20 µg/mL proteinase K solution for 15 min. Then, the sections were incubated with 100 μL of 0.3% H_2_O_2_ for 5 min and incubated by equilibration buffer and terminal deoxynucleotidyl transferase to inactivate endogenous peroxidase. Then, anti-digoxigenin–peroxidase conjugate was employed to incubate the sections. Finally, the utilization of diaminobenzidine demonstrated peroxidase activity and the slices were counterstained with hematoxylin. The results were scored semi-quantitatively at magnification ×400 with Image Pro Plus software.

### 2.6. Analysis of Immunofluorescence Staining

The tissue samples were deparaffinized and rehydrated, then treated with citrate buffer solution (0.01 M, pH 6.0) for 20 min [24]. After washing with PBS for three times, the sections were incubated with 1% bovine serum albumin (BSA) for 10 min to block non-specific binding at room temperature. Thereafter, the liver sections were incubated at 4°C overnight with primary antibodies including rabbit anti-BNIP3 antibody (1:200 dilution) and rabbit anti-LC3 antibody (1:200 dilution) followed by secondary antibody for 30 min. After washing with PBS for three times, the slices were incubated with DyLight 488 after 12 h at 37 °C. Then, nuclear structures were exposed to 4,6 diamidino-2-phenylindole (DAPI) staining. Images were captured by a Leica microscope (Leica TCS SP8, Solms, Germany).

### 2.7. Estimation of Lipid Peroxidation and Biochemical Parameters

The levels of GSH and MDA were analyzed using commercial reagent kits according to the manufacturer’s protocols. Samples containing lipid peroxidation were mixed with Thibabituric Acid (TBA) to form red mixture. The absorbance of the sample was detected at 532 nm. Frozen tissues were homogenized and centrifuged (3500 r for 5 min) to separate the supernatant. Hepatic GSH were react with 5,5′-Dithiobis-(2-nitrobenzoic acid) (DTNB) solution to produce a yellow compound. 

Serum ALT and AST were detected spectrophotomerically using commercial reagent kits (Jiancheng, Nanjing, China).

### 2.8. Measurement of Serum Inflammatory Markers TNF-α and IL-1β

The concentrations of TNF-α and IL-1β in serum were detected by commercial ELISA kits measured at 450 nm for absorbance following the manufacturer’s instructions.

### 2.9. Western Blotting Analysis 

The total protein of liver tissues was prepared using RIPA buffer (1:10, g/v). The protein concentration of liver tissues was quantitative using the tissue BCA protein assay kit (Beyotime Biotechnology, China) based on the manufacturer’s instruction. The protein samples were loaded in 12% SDS-PAGE gel and transferred to PVDF membrane. After blocking in 5% non-fat dry milk in Tris-buffered saline-T (TBST, 0.1% Tween-20 in TBS) for 1 h, the membranes was incubated overnight at 4 °C with primary antibodies including Bax (1:2000 dilution), Bcl-2 (1:2000 dilution), caspase 3 (1:2000 dilution), cleaved caspase 3 (1:1000 dilution), mTOR (1:500 dilution), p-mTOR (1:1000 dilution), Akt (1:1000 dilution), p-Akt (1:1000 dilution), p62 (1:500 dilution), LC3 II / I (1:1000 dilution), and β-actin (1:1000 dilution). After washing with TBST three times, the membranes were incubated for 1.5 h with the secondary antibodies. The protein bands were measured using Quantity One software (Bio-Rad Laboratories, Hercules, CA, USA).

### 2.10. Statistical Analysis and Data Presentation

All data are expressed as mean ± SD. The statistical significance of mean values was assessed using Student *t*-test and one-way ANOVA, followed by a Bonferroni post hoc test. Statistics were performed using GraphPad Prism 6.0.4 software. *p* < 0.05, *p* < 0.01 or *p* < 0.001 were defined as statistically significant.

## 3. Results

### 3.1. α-MG Restores APAP-Induced Liver Histopathological Changes

**Figure 2:**
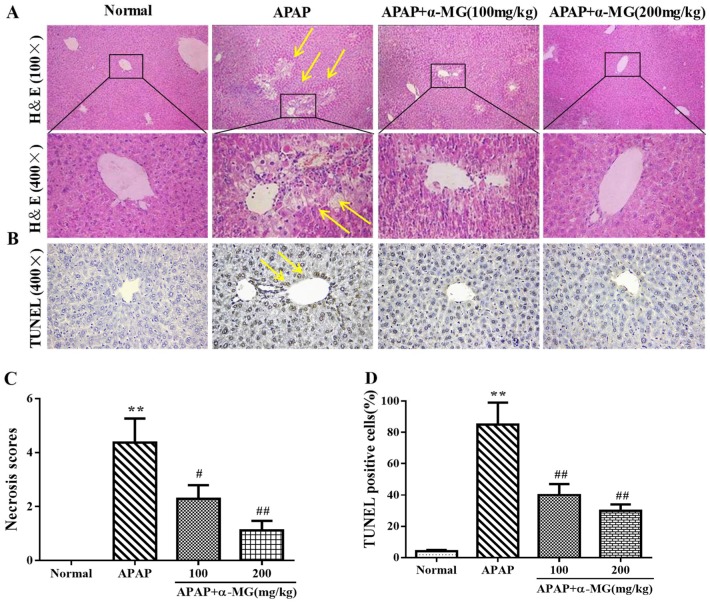
Inhibition of APAP-induced acute liver injury by α-MG. Representative sections of liver stained with hematoxylin and eosin (H&E), (100×, 400×) for histopathological observations. Hepatocellular necrosis was marked by arrows (**A**). The degrees of damage were accessed by necrosis scores (**C**). Analysis of apoptosis in mice was evaluated by TUNEL staining (**B**). Representative liver sections were stained with TUNEL (400×). The TUNEL positive cells percentage is shown in (**D**); All data are expressed as mean ± S.D., *n* = 8. ** *p* < 0.01 vs. normal group; ## *p* < 0.01 or # *p* < 0.05 vs. APAP group.

To explore the beneficial role of α-MG against APAP hepatotoxicity, we performed a histological evaluation of the liver injury. Histology of liver tissue in the control group showed normal hepatic morphology with central-rounded well-defined vesicular nuclei and cytoplasm. Hepatocytes were located around central vein and separated by blood sinusoids lined by endothelial cells. Liver from mice treated with APAP displayed sustained liver injury characterized by severe necrosis, sinusoidal congestion, hepatocyte swelling, inflammatory infiltration, and hydropic degeneration. By contrast, administration of α-MG remarkably improved histopathological changes elicited by APAP, especially at the high dosage of 200 mg/kg (Figure 2A). Furthermore, the histopathological changes in each section were accessed, and scores represented the approximate necrotic extent around the central vein areas (Figure 2C). 

### 3.2. Ameliorating Effects of α-MG against APAP-Induced Aberrant Transaminase

**Figure 3:**
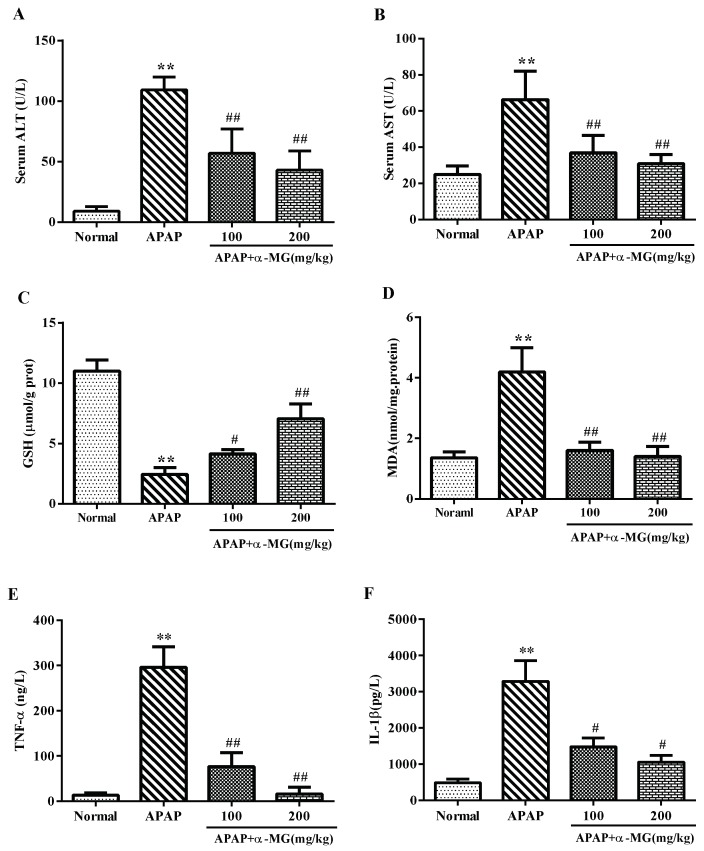
Effects of α-MG on serum enzyme activity, oxidative stress, and inflammatory responses. ALT (**A**); AST (**B**); GSH (**C**); MDA (**D**); TNF-α (**E**) and IL-1β (**F**) levels from mice in each experimental group were determined by commercial kits. All data are expressed as mean ± S.D., *n* = 8. *** *p* < 0.001 or ** *p* < 0.01 vs. normal group; ### *p* < 0.01 or ## *p* < 0.01 vs. APAP group.

As shown in Figure 3A, the serum levels of ALT and AST were significantly increased in APAP-treated group compared with the normal group (*p* < 0.001, *p* < 0.01), confirming the induction of severe intoxication. In the APAP-group treated with α-MG, the levels of two aminotransferase enzymes were significantly reduced as compared with the APAP group, respectively, indicating that α-MG supplementation protected the liver from APAP toxicity.

### 3.3. Inhibitory Effects of α-MG on Oxidative Stress

The oxidative stress induced by APAP is normally detoxified by the enzymatic antioxidant defense system. The content of GSH was much lower in APAP group compared with the normal group (*p* < 0.001). α-MG supplementation inhibited the depletion of hepatic GSH content after APAP injection in a dose-dependent manner (Figure 3C) (*p* < 0.01). Meanwhile, the APAP-treated mice exhibited the significantly production of MDA compared with normal mice (*p* < 0.01). Interestingly, α-MG dramatically suppressed the APAP-mediated excessive production of MDA (Figure 3D) (*p* < 0.01). In brief, α-MG treatment may reduce the oxidative stress and restore endogenous antioxidant system to prevent APAP-induced hepatotoxicity.

### 3.4. α-MG Inhibits APAP-Inducible Inflammation

Tissue inflammation is frequently activated when hepatocytes were injured. Hence, to elucidate the possible inhibitory effect of α-MG on inflammation-related factors in the liver, we performed ELISA measurements of these factors using serum samples with or without α-MG administration. The contents of TNF-α and IL-1β 24 h after APAP injection increased significantly as compared with that in the normal group without APAP injection (*p* < 0.01 or *p* < 0.01). As expected, α-MG inhibited APAP-stimulated elevation of TNF-α and IL-1β in a dose-dependent manner (Figure 3). At the α-MG dose of 200 mg/kg, the level of TNF-α was similar to that of the normal group. Thus, α-MG drastically reduced the release of pro-inflammatory cytokines including TNF-α and IL-1β by APAP injection, suggesting that α-MG may have anti-inflammatory potential too.

### 3.5. Inhibitory Effects of α-MG on Apoptotic Molecular Expression

We evaluated the protective role of α-MG against APAP-induced hepatic apoptosis using TUNEL staining kits (Figure 2B). Compared with the normal group, large number of apoptotic cells was visualized in APAP group (*p* < 0.01). The percentage of apoptotic cells in APAP and α-MG co-treatment group was remarkably decreased (*p* < 0.01), respectively. 

**Figure 4:**
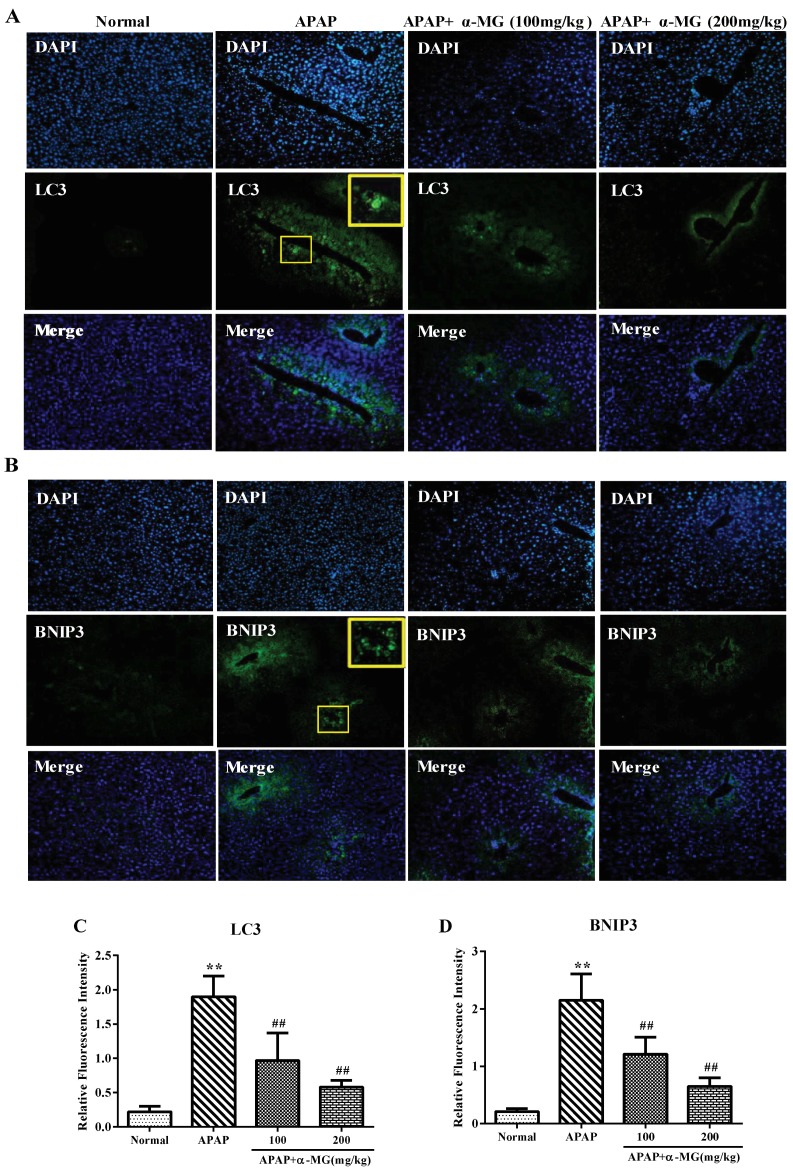
Inhibition of APAP-induced autophagy by α-MG. The expression of LC3 (**A**) and BNIP3 (**B**) in liver tissues from each experimental group was determined by immunofluorescence; The fluorescence intensity of LC3 (**C**) and BNIP3 (**D**) (green fluorescent) was accessed by photodensitometry. Representative immunofluorescence images were taken at 200×. DAPI (4′,6-diamidino-2-phenylindole4, blue) was used as a nuclear counterstain. All data are expressed as mean ± S.D., *n* = 8. ** *p* < 0.01 vs. normal group; ## *p* < 0.01 vs. APAP group.

BNIP3, a member of the Bcl-2 homology 3 (BH3) domains-only Bcl-2 families, interacts with Bcl-2 and Bcl-XL to induce cell apoptosis, death and autophagy [25]. The protein expression level of BNIP3 after a single injection of APAP was significantly increased as compared with that in the normal group. Amount of BNIP3 punctate was declined by α-MG treatment, respectively. These findings suggest that α-MG may inhibit the apoptosis caused by APAP injection (Figure 4).

**Figure 5:**
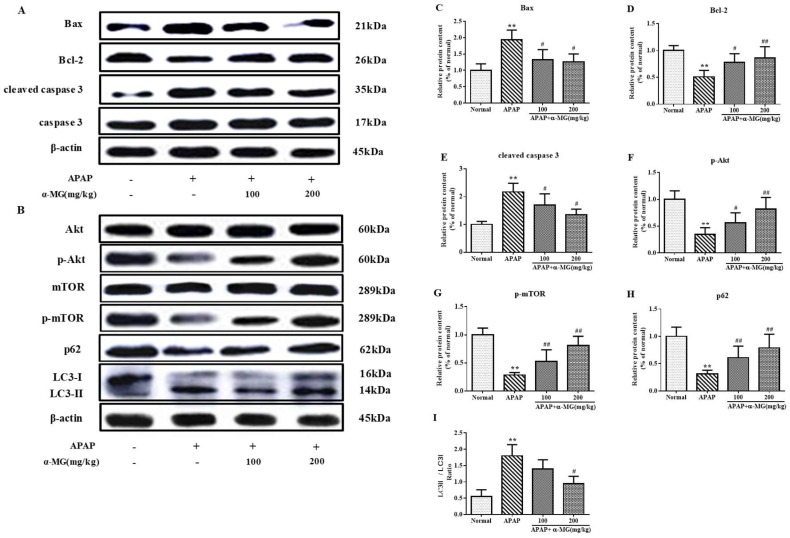
Suppression of apoptotic and autophagic pathway by α-MG using Western blotting analysis. The intensity of Bax, Bcl-2, caspase 3, cleaved caspase 3, Akt, p-Akt, m-TOR, p-m-TOR, p62 and LC3 II /LC3 I ratio were standardized to that of β-actin (**A**,**B**); Quantitative analysis of scanning densitometry for Bax (**C**); Bcl-2 (**D**); cleaved caspase 3 (**E**); p-Akt (**F**); m-TOR (**G**); p62 (**H**) and LC3 II /LC3 I ratio (**I**); were performed. All data are expressed as mean ± S.D., *n* = 8. ** *p* < 0.01 vs. normal group; ## *p* < 0.01 or # *p* < 0.05 vs. APAP group.

To explore the effect of α-MG administration on apoptosis-related signals, pro-apoptotic factor Bax, anti-apoptotic factor Bcl-2, and caspase 3 protein activities in the liver samples were analyzed by Western blotting analysis. The increase in Bax protein expression and decrease in Bcl-2 protein expression were observed in the liver tissues in APAP-injected mice. α-MG supplementation significantly reversed these changes at a dose-dependent manner (Figure 5A). To gain a better understanding of the underlying mechanism, we measured the anti-apoptotic effect of α-MG on APAP-induced liver injury. The protein expression level of cleaved caspase 3 was analyzed. Obviously, the expression of cleaved caspase 3 was increased by APAP injection and α-MG administration attenuated this elevation. Collectively, these results suggest that α-MG may effectively limit the APAP-induced acute liver injury by regulating apoptosis-related signals.

### 3.6. Suppression of Molecular Expression in Autophagic Pathway in Mice by α-MG

In the liver tissues obtained from mice injected with APAP, the fluorescence intensity of LC3 (an autophagy diagnostic indicators) displayed a punctate pattern around the central veins, where autophagy is gathered. Indeed, the increased expression of LC3 punctate after APAP injection indicated that APAP-induced hepatotoxicity stimulated the genesis of an autophagy-related morphological change process. Interestingly, the increased fluorescence intensity of LC3 after APAP injection was significantly decreased after α-MG treatment, particularly in the dosage of 200 mg/kg (*p* < 0.01), which might reflect a suppression of excessive autophagy.

Additionally, the hepatic protein expression levels of Akt, p-Akt, mTOR, p-mTOR, LC3 and p62 were determined by the appropriate Western blotting analysis to assess the influence of α-MG on APAP-caused autophagy. As shown in Figure 5B, p-Akt and p-mTOR were reduced in APAP group but elevated by α-MG administration. Further results showed a significant increase in the LC3 II/LC3 I ratio after APAP exposure in comparison to the normal group. Conversely, the LC3 II/I ratio in APAP/α-MG co-administration group significantly declined compared with the APAP-only group. Moreover, the level of p62/SQSTM1 expression declined in mice treated with APAP, whereas α-MG administration inhibited degradation of p62/SQSTM1 protein. These findings suggest that the overactivation of autophagy by APAP injection may be inhibited by α-MG treatment.

## 4. Discussion

The liver is the primary organ with metabolic function and can be intoxicated by infections, drug exposure, excessive alcohol drinking, accidental food poisoning, radiation damage, environmental pollutants, and several other factors [26]. APAP is the most frequent causative agent when misused at a single overdose in animals and human [27]. It has been established that the overdose of APAP is bio-transformed to NAPQI, which binds covalently to tissue macromolecules and oxidizes lipids, leading to hepatotoxicity [28].

Lipid peroxidation is known to cause membrane disruption, resulting in the loss of membrane integrity and leakage of microsomal enzymes [29]. Generally, GSH plays a key role in mitochondrial antioxidant defense and its depletion induced by NAPQI is an important event in APAP-induced toxicity [30]. In addition, MDA as an end-product of lipid peroxidation is usually used as a bio-indicator of oxidative stress. In the present work, administration of α-MG dramatically increased GSH stores, and decreased MDA levels as well as excessive ROS, consistent with the antioxidant activity and free radical scavenging effect of α-MG reported in other experimental models [31]. According to presented findings and previous reports, it is speculated that α-MG may directly suppress oxidative stress and the accumulation of ROS to detoxification of overdose APAP injection. 

It has been demonstrated that the severity of the hepatotoxicity and the increased necrotic area may be related to the increased release of inflammatory mediators such as TNF-α and IL-1β in APAP-induced ALI [32]. TNF-α and IL-1β, the two major pro-inflammatory cytokines, stimulated the matrix metalloproteinases, and ultimately resulted in liver failure [33]. Our data regarding hepatic protein expressions of these pro-inflammatory cytokines clearly indicated that inflammation was pronouncedly mitigated by α-MG treatment. The findings in our work are consistent with a previous report that α-MG suppressed the overproduction of IL-1β, IL-6, and TNF-α in microglia-mediated neuroinflammation [34].

Apoptosis of hepatocytes is directly involved in hepatotoxicity in the pathogenesis of liver disease. The Bcl-2 protein family mainly consists of pro-apoptotic protein Bax and anti-apoptotic protein Bcl-2, which play important roles in the determination of apoptosis in response to many pathological processes [35]. Anti-apoptotic Bcl-2 family members attenuate the release of killer proteins, protect mitochondrial integrity and prevent apoptosis [36]. Caspase-3, a member of the caspase family of 14 aspartate-specific cysteine proteases, also plays vital roles in the apoptotic program. Evidence demonstrated APAP-induced translocation of Bcl-2 family proteins [37] and the activation of caspase 3 [38]. In addition, BNIP3 caused apoptosis and promoted Bax/Bad-dependent release of pro-apoptotic mediators [39]. Present results revealed that APAP injection downregulated Bcl-2 and upregulated Bax, and cleaved caspase 3 protein expression in hepatocytes, which was in line with our previous studies [23]. Importantly, supplementation of α-MG partially maintained the balance between Bax and Bcl-2, resulting in the prevention of mitochondrial dysfunction caused by APAP. In fact, our results were supported by a recent reports by Luo et al., who reported that α-MG exerted anti-apoptotic effects on high-glucose-induced vascular endothelial cell dysfunction via upregulation of Bcl-2 and downregulation of Bax protein expression [40].

The current work focused on exploring the relationship between apoptosis and autophagy after APAP injection in mouse models. Autophagy is a highly conservative process in evolution [41], and is responsible for degrading or recycling dysfunctional cellular organelles and bio-macromolecules in all living cells [42]. Although physiological levels of autophagy are essential during various stress conditions, excessive autophagy may cause autophagy-dependent cell death [43]. Autophagic process is an early adaptive response process, placing in the upstream of apoptosis [44]. Specifically, autophagic activation may result in autophagic cell death and execution of apoptotic cell death via regulating Bcl-2 family proteins. Furthermore, Bcl-2 proteins may bind to autophagosome-related genes to inhibit autophagy. Therefore, both autophagy and apoptosis may be regulated by Bcl-2 family proteins [45]. In the present work, α-MG treatment subdued partially the overexpression of autophagy caused by APAP injection. A recent report, however, confirmed that α-MG-induced autophagic cell death in brain cancer cells was mediated by the activation of the AMP-activated protein kinase (AMPK) pathway, suggesting that α-MG may exert bidirectional effects by regulating autophagy-related molecules [46]. 

The Akt/mTOR pathway is an important signaling pathway in regulating the intracellular homeostasis. It is reported that the activation of Akt signal pathway further can facilitate liver repair and regeneration [47] and attenuates APAP-induced liver injury [48]. A recent study has shown that activation of Akt/mTOR signaling pathway is essential in the inhibition of autophagy [49]. Briefly, Akt activates the downstream mTOR signaling pathways and mTOR inhibits autophagy. In the process of autophagosome formation, lipid conjugation of free LC3 I is transferred to the autophagic membrane-associated LC3 II, which results in the elevated LC3 II level and the activation of autophagy [50]. Notably, LC II’s accumulation may be a consequence either of autophagy activation or inhibition of downstream autophagy steps [51]. Therefore, assessment of the level of p62/SQSTM1, an autophagy adaptor molecule, is essential for determining the production of autophagosomes [52]. It is notable that LC3 protein, a commonly used marker of autophagosome formation, is associated with a number of autophagosomes [53]. In the present study, immunofluorescence staining of LC3 puncta was employed to assess autophagy levels after APAP injection and α-MG administration. The fluorescence intensity of LC3 significantly increased in liver tissue after APAP injection, whereas α-MG reversed this increase in a dose-dependent manner. Meanwhile, Western blot analysis was performed to confirm this variation of LC3 protein expression in the liver tissues. As expected, APAP injection aggravated liver damage, evidenced by the increase in autophagosome formation and LC3 II/LC3 I ratio. However, α-MG treatment partially inhibited the overexpression of LC3 II, accompanied by the increased protein expression of LC3 I, indicating that α-MG may protect APAP-induced liver injury by modulating autophagy-related proteins.

**Figure 6:**
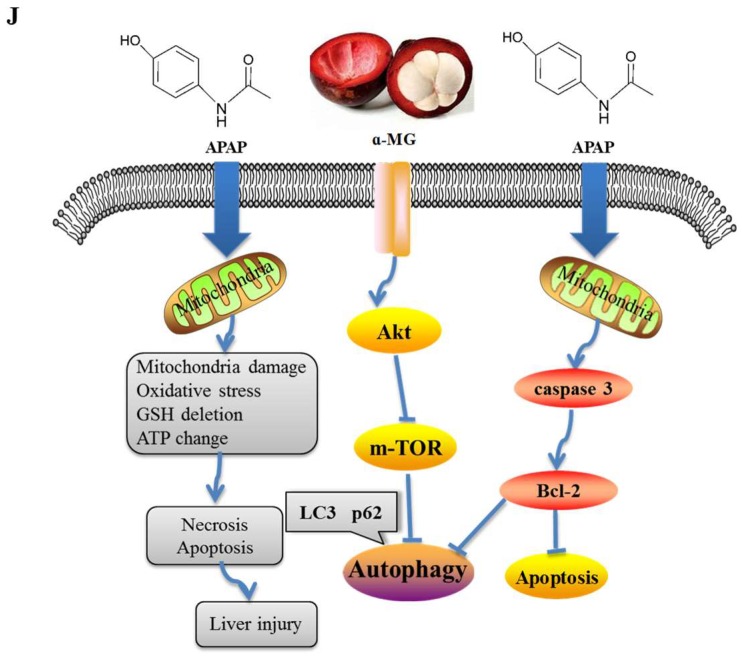
Model of action of APAP and α-MG.

Taken all together, to the best of our knowledge, this work is the first report to demonstrate that α-MG treatment alleviates adverse effects of overdose APAP-induced hepatic injury in mice partly via the restoration of anti-oxidative activity and modulation of inflammation, apoptosis and autophagy. Importantly, we found that the protective effect of α-MG on APAP-induced ALI might be due to the alterations in Akt/mTOR signaling pathway in the liver (Figure 6). However, the usage of antagonists and knockouts to confirm the hepatoprotective effect of α-MG has not yet been proven. Further studies may be undertaken to demonstrate that these changes are responsible for the protective effect of α-MG against APAP-induced hepatic injury. More studies are warranted to explore the beneficial effect of α-MG in other liver injury model by pharmacological screening in experimental animals.
